# Evaluation of the promoter region polymorphism (5-*HTTLPR*) in the serotonin transporter gene in females with postpartum depression

**DOI:** 10.3892/etm.2014.2043

**Published:** 2014-10-31

**Authors:** XIAOLI ZHANG, LIN WANG, FENGHUA HUANG, JIAFU LI, LI XIONG, HAN XUE, YUANZHEN ZHANG

**Affiliations:** 1 Department of Obstetrics and Gynecology, Zhongnan Hospital of Wuhan University, Wuhan, Hubei 430071, P.R. China; 2 Department of Histology and Embryology, Medical College of Wuhan University, Wuhan, Hubei 430071, P.R. China; 3 Department of Neurology, Zhongnan Hospital of Wuhan University, Wuhan, Hubei 430071, P.R. China; 4 Department of Clinical Laboratory, Zhongnan Hospital of Wuhan University, Wuhan, Hubei 430071, P.R. China

**Keywords:** postpartum depression, 5-*HTTLPR*, serotonin transporter, gene polymorphism

## Abstract

The aim of the present study was to investigate the association between polymorphism in the serotonin transporter (5-HTT) gene-linked polymorphic region (5-*HTTLPR*) in the promoter region of the 5-HTT gene and the pathogenesis of postpartum depression (PPD). Blood samples were collected from 120 female patients with PPD and 140 age-matched normal controls. Polymerase chain reaction analysis was performed to detect the 5-*HTTLPR* polymorphism in these subjects, and the genotype and allele frequencies were compared between the two groups. The disease severity was evaluated using the Hamilton Depression Rating Scale (HAMD) score. The results showed that the frequency of the homozygous long/long (L/L) genotype was significantly lower in the PPD group than that in the control group; by contrast, the frequencies of the heterozygous long/short (L/S) and homozygous S/S genotypes were similar for the two groups, without significant differences. No significant differences were observed in the L and S allele frequencies between the two groups. Furthermore, compared with the L/S heterozygous and S/S homozygous genotypes, patients with PPD with the L/L homozygous genotype had a significantly lower HAMD score. The present results suggest that female patients with PPD carrying the homozygous L/L genotype may be less susceptible to depressive symptoms and that the L/L genotype may be associated with the reduced occurrence of PPD. These findings provide a theoretical basis for the clinical diagnosis and treatment of PPD.

## Introduction

Postpartum depression (PPD) is a mood disorder in females that usually presents within the first 4–6 weeks after childbirth; the condition is clinically characterized by depression, sadness, frustration, crying, irritability, restlessness and even suicidal tendencies (1). According to a previous meta-analysis, PPD is prevalent worldwide, and 10–15% of females may be affected (2). In addition to the general symptoms of depression, PPD can, in certain cases, be associated with a disturbance of consciousness, psychotic symptoms and Schneider’s symptoms, which can only be alleviated by regular treatments (3,4). It has been acknowledged that PPD is induced by biological, psychological and/or social factors; bio-genetics in particular is closely linked with the mental illnesses (5,6).

Recent genetic studies have shown that dysfunction of the 5-hydroxytryptamine (5-HT) system is the key factor in the development of depression (7,8). Accordingly, genes associated with the synthesis, release, uptake and metabolism of 5-HT could become candidates for studies on the pathogenesis of depression. The serotonin transporter (5-HTT) plays an important role in the re-uptake of 5-HT following release, and therefore is the target for the majority of antidepressants (9,10). A recent study indicated that the transcriptional activity of the human 5-HTT gene is regulated by the 5-HTT gene-linked polymorphic region (5-*HTTLPR*) (11), with long (L) and short (S) alleles. The L allele in 5-*HTTLPR* is associated with higher transcriptional efficiency of the promoter compared with the S allele. The mRNA transcription and protein expression levels of 5-HTT are higher in individuals with a homozygous L/L genotype than those with S/S genotypes (12).

To date, the association between 5-*HTTLPR* gene polymorphism and PPD has not been fully established, and there are few studies focusing on the association between the genetic polymorphism and the clinical characteristics. In the present study, the 5-*HTTLPR* status in the 5-HTT gene was evaluated in order to establish whether it had an association with PPD pathogenesis in Han female patients.

## Materials and methods

### Patients

A total of 120 Han female patients with PPD, aged 20–35 years (mean age, 28.57±6.8 years), with a course of disease ranging between six weeks and six months, were enrolled in the present study. The patients had all been admitted to the Zhongnan Hospital of Wuhan University (Wuhan, China) between 2008 and 2012. The patients had been diagnosed with PPD according to the diagnostic criteria for depression (for PPD, occurring within 6 months after delivery) from the Chinese Classification of Mental Disorders (13), as well as the Hamilton Depression Rating Scale (HAMD) score (24-item version). An additional 140 Han females with similar social background were included as normal controls, aged 20–35 years (mean age, 26.18±7.2 years). None of the subjects or their parents were involved in drug abuse and/or addiction, nor had a history of mental illness, serious physical illness or organic brain diseases. Prior written and informed consent was obtained from every patient and the study was approved by the Ethics Review Board of the Zhongnan Hospital of Wuhan University.

### Blood sample collection

Peripheral blood samples from the subjects in the PPD and control groups were collected under fasting conditions in the morning. A total of 100 μl EDTA (0.5 mol/l; pH 8.0) was added for anticoagulation, and these blood samples were stored at −80°C until use.

### DNA extraction and polymerase chain reaction (PCR) analysis

**Figure 1:**
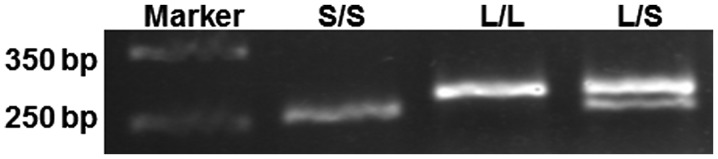
Genotype analysis of 5-*HTTLPR* polymorphism in the 5-hydroxytryptamine transporter promoter region with polymerase chain reaction amplification. L, long; S, short.

Genomic DNA was extracted using a whole blood genomic DNA extraction kit (Qiagen GmbH, Hilden, Germany), according to the manufacturer’s instructions, and then analyzed with 0.8% agarose gel electrophoresis. Amplification primers for the promoter region of the 5-HTT gene were as follows: Forward primer, 5′-GCGCTCCTGCATCCCCCATTA-3′; and reverse primer, 5′-GGGATGCGGGGGAATACTGGT-3′. 5-*HTTLPR* polymorphism in the 5-HTT promoter region was assessed using PCR amplification. A total of 25 μl PCR reaction solution was prepared by mixing 10X PCR reaction buffer [containing 2.0 mmol/l (NH_4_)_2_SO_4_ and MgCl_2_; Invitrogen Life Technologies, Carlsbad, CA, USA], 0.16 μmol/l each primer, 1.5 units Taq enzymes and 160 μmol/l deoxyribonucleotide triphosphates. The PCR conditions were as follows: Denaturation at 95°C for 2 min, followed by 35 cycles of 95°C for 60 sec, 62°C for 60 sec and 72°C for 60 sec, and then extension at 72°C for 10 min. The PCR products were separated by 2% agarose gel electrophoresis, and the gel was visualized and analyzed by a Bio-Rad image analysis system (Bio-Rad, Hercules, CA, USA). Two different allelic fragments with the lengths of 297 bp (L) and 253 bp (S) were detected among the PCR products, and the genotype was determined accordingly (Fig. 1) (14).

### Statistical analysis

Statistical analysis was performed using SPSS 13.0 software (SPSS, Inc., Chicago, IL, USA). A χ^2^ test of goodness of fit was used to test the Hardy-Weinberg (H-W) equilibrium in the samples. P<0.05 was considered to indicate a statistically significant difference.

## Results

### H-W equilibrium test of the 5-HTTLPR genotype distribution

To investigate the representativeness of the samples, the H-W equilibrium, with the respect to the 5-*HTTLPR* polymorphism, was examined in the population of the PDD and control groups. The results showed that the genotype frequency distribution of the 5-*HTTLPR* polymorphism in the enrolled population was in H-W equilibrium, and no significant differences were found between the observed and expected values (P>0.05) (Table I). These results suggest that the enrolled population was a good representative sample.

### Comparison of 5-HTTLPR polymorphism genotype distribution between the PPD and control groups

**Figure 2:**
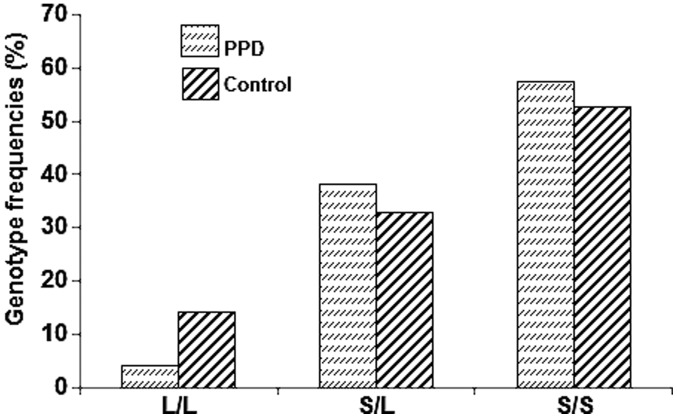
Comparison of the 5-*HTTLPR* genotype frequencies between the PPD and control groups. The frequency of the L/L genotype was significantly lower in the PPD group than that in the control group, while the frequencies of the L/S and S/S genotypes were similar for these two groups. PPD, postpartum depression; L, long; S, short.

**Figure 3:**
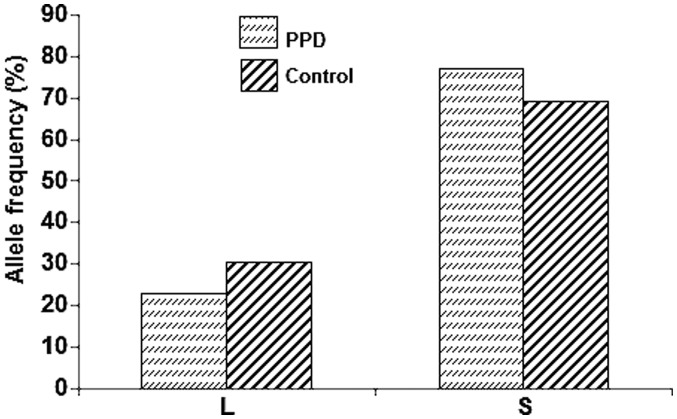
Comparison of the 5-*HTTLPR* allele frequencies between the PPD and control groups. No significant differences were observed in the L and S allele frequencies between the PPD and control groups. PPD, postpartum depression; L, long; S, short.

To investigate the association between 5-*HTTLPR* gene polymorphism and PPD, the genotype frequencies were examined and compared between the PPD and control groups. The results showed that the frequency of the homozygous L/L genotype was significantly lower in the PPD group (4.17%) than that in the control group (15.83%) (χ^2^, 6.854; P=0.032). By contrast, the frequencies of the heterozygous L/S and homozygous S/S genotypes were similar in the two groups, without significant differences (Table II and Fig. 2). In addition, no significant differences in the frequencies of the L and S alleles were found between the PPD and control groups (χ^2^, 3.226; P=0.072) (Table III and Fig. 3). These results suggest that the L/L homozygous genotype may be associated with the reduced occurrence of PPD.

### Association between 5-HTTLPR polymorphism genotype and HAMD score in PPD

To further investigate the role of the 5-*HTTLPR* polymorphism genotypes in PPD, the disease severity was evaluated using the HAMD score. The results showed that, compared with the L/S heterozygous and S/S homozygous genotypes, patients with PPD carrying the L/L homozygous genotype had a significantly lower HAMD score (P<0.001) (Table IV). These results suggest that homozygous L/L carriers with PPD may be less susceptible to the depressive symptoms.

## Discussion

PPD was first described by Pitt in 1968 (15). Epidemiological studies show that patients with PPD often exhibit seriously depressed mood following delivery, including various psychotic and mood disorders (16,17. PPD jeopardizes the physical and mental health of the parturient females, as well as their infants. Under certain circumstances, postpartum psychosis can also be induced, causing a substantial economic burden for families and society. The pathophysiology of PPD is complex, and the mechanism is still yet to be thoroughly clarified. It is believed that the pathogenesis and development of PPD involve a variety of internal and external factors (18).

In recent years, with the rapid development of human genomics studies, attempts have been made to elucidate the association between genetic polymorphisms and PPD susceptibility (19,20). It has been shown that the neurotransmitter 5-HT is involved in regulating a number of physical and mental activities, and plays an important role in the pathogenesis of depression (21–22). Furthermore, the re-uptake of 5-HT is regulated by the 5-HTT, which is widely distributed on the presynaptic membranes in the central nervous system (24). Pre-clinical and clinical studies have therefore focused on this target for the treatment of depression (25,26).

The human 5-HTT gene (also known as *SLC6A4*) is located on chromosome 17q11.1-q12 and contains 14 exons. The 5-HTT gene-linked polymorphic region (5-*HTTLPR*) has been recognized as one of the functional polymorphism sites of the 5-HTT gene (27). There are three genotypes, L/L, L/S and S/S, for 5-*HTTLPR* polymorphism. A number of studies have shown that the S allele could be the susceptible factor for depression, while the genotype of L/L would be protective against depression (28–30). By contrast, other studies have revealed that the frequency of the L allele in patients with depression is considerably higher than that in the control group, indicating an association between the L allele and the pathogenesis of depression (31,32). Other studies, however, have speculated that 5-*HTTLPR* is not associated with depression, or at least is not the major risk factor (33–37).

In the present study, it was found that the L and S allele frequencies in Han female patients with PPD were similar to those in normal controls. The S allele was predominant in normal Han females (69.64%), with the S/S genotype accounting for 52.86% and the L/L genotype for 15.83% of the genotypes in the group. This was consistent with previous studies concerning the genotype distribution and allele frequencies in the Han population (38,39). Notably, the present results indicated that the frequency of the L/L homozygous genotype was significantly lower in the patients with PPD (4.17%) than that in the normal control females (15.83%). In addition, the HAMD score from the patients with PPD with the L/L homozygous genotype (27.8±2.39) was markedly lower than that either from the L/S (41.4±2.26) or the S/S (45.4±3.39) carriers, indicating less severe depressive symptoms. These results suggest that the L/L homozygous genotype could exert protective effects against the onset and development of PPD, reducing the susceptibility of patients to the disease.

In conclusion, the present results revealed the association between the 5-*HTTLPR* polymorphism and the pathogenesis of PPD in the Han population. Specifically, it was shown that the L allele in 5-*HTTLPR* was associated with a reduced susceptibility to PPD. These findings provide a theoretical basis for the clinical diagnosis and treatment of PPD. Due to the limited sample size of the present study, further studies with expanded samples are still required to validate the present results and to fully address the role of 5-*HTTLPR* polymorphism in PPD.

## Figures and Tables

**Table I:** Hardy-Weinberg equilibrium test of the 5-*HTTLPR* genotype distribution in the PPD and control groups.

Group	Genotype	Observed value	Expected value	χ^2^	P-value
PPD	L/L	5	6.51		
	L/S	46	42.89		
	S/S	69	70.59	0.612	>0.05
Control	L/L	19	12.90		
	L/S	47	59.09		
	S/S	74	67.90	2.884	>0.05

PPD, postpartum depression; L, long; S, short.

**Table II:** Comparison of the 5-*HTTLPR* genotype frequencies between the PPD and control groups.

		5-*HTTLPR* genotype frequencies		
		
Group	N	L/L, % (n)	L/S, % (n)	S/S, % (n)
PPD	120	4.17 (5)^a^	38.33 (46)	57.50 (69)
Control	140	15.83 (19)	39.17 (47)	52.86 (74)
Total	260	9.23 (24)	35.77 (93)	55.00 (143)

a Compared with the control group, χ^2^=6.854 and P=0.032.

PPD, postpartum depression; L, long; S, short.

**Table III:** Comparison of the 5-*HTTLPR* allele frequencies between the PPD and control groups.

		5-*HTTLPR* allele frequencies	
		
Group	N	L, % (n)	S, % (n)
PPD	240	23.33 (56)	76.67 (184)
Control	280	30.36 (85)	69.64 (195)
Total	520	27.12 (141)	72.88 (379)

No significant differences were found in the frequencies of the L and S alleles between the PPD and control groups (χ^2^=3.226 and P=0.072). PPD, postpartum depression; L, long; S, short.

**Table IV:** HAMD scores for patients with postpartum depression with different genotypes.

Genotype	N	HAMD score	F	P-value
L/L	5	27.8±2.39	78.33	<0.001
L/S	46	41.4±2.26		
S/S	69	45.4±3.39		

HAMD, Hamilton Depression Rating Scale; L, long; S, short.
